# Case Report: Renal artery aneurysm rupture, axillary artery pseudoaneurysm, and catastrophic intraperitoneal hemorrhage caused by fibromuscular dysplasia with the involvement of multiple arterial beds

**DOI:** 10.3389/fsurg.2026.1732288

**Published:** 2026-03-11

**Authors:** Haihua Zhou, Shi Sheng, Yun You, Jian Wang

**Affiliations:** Department of Vascular Surgery, Union Hospital, Tongji Medical College, Huazhong University of Science and Technology, Wuhan, China

**Keywords:** aneurysm, axillary artery, fibromuscular dysplasia, intraperitoneal hemorrhage, pseudoaneurysm, renal artery

## Abstract

We report an unusual case of fibromuscular dysplasia (FMD) with multisite involvement into the intracranial, carotid, vertebral, internal mammary, visceral, renal, iliac, axillary, and upper extremity arteries in a 30-year-old woman. The patient presented with a spontaneous rupture of a renal artery aneurysm and an expanding axillary artery pseudoaneurysm. The diagnosis of the multifocal FMD was based mostly on the classical strings-of-beads angiographic appearance of the upper extremity, visceral, vertebral, carotid, and internal mammary arteries. A coil embolization procedure was successfully performed to treat the ruptured renal aneurysm. In addition, open surgical repair of the pseudoaneurysm was uneventfully achieved using direct arterial suture. Unfortunately, the patient suffered from recurrent intraperitoneal hemorrhage, declined further endovascular interventional or open surgical procedures, and eventually died from hemorrhagic shock and multiple organ failure. To our knowledge, this is the first case in the literature describing a rare and severe FMD involving multiple arterial beds in the regions of the head, neck, chest, abdomen, and pelvis, which adversely caused renal artery aneurysm rupture, axillary artery pseudoaneurysm, and catastrophic intraperitoneal hemorrhage.

## Introduction

Fibromuscular dysplasia (FMD) is an idiopathic, segmental, non-inflammatory, non-atherosclerotic arteriopathy of unknown etiology that occurs most commonly in middle-aged women (1). FMD predominantly affects small and medium-sized arteries throughout the body, particularly the renal, carotid, and vertebral arteries, but it may also involve other arterial beds such as the intracranial, visceral branch, iliac, upper extremity, and even coronary arteries. However, an FMD involving multiple arterial beds in the regions of the head, neck, chest, abdomen, and pelvis is rare. Specifically, FMD may manifest as stenosis, aneurysm, dissection, occlusion, and arterial tortuosity. Alternating areas of arterial stenosis and dilation are classically described as a “string-of-beads” appearance on angiography (2). Hemodynamic changes related to the stenotic, occlusive, and aneurysmal segments may cause thrombus deposition with distal embolization and/or end-organ ischemia or infarction. The most worrisome vascular complications of the disease are aneurysm rupture and pseudoaneurysm formation; however, artery aneurysm or pseudoaneurysm rupture secondary to FMD is also exceedingly rare. This case report represents a rare and severe presentation of FMD with multisite involvement of the intracranial, carotid, vertebral, internal mammary, visceral branch, renal, iliac, axillary, and upper extremity arteries, which adversely cause renal artery aneurysm rupture and axillary artery pseudoaneurysm formation as well as massive intraperitoneal hematoma.

## Case report

A 30-year-old woman presented to the emergency department of our hospital with pain and swelling in the left upper extremity, swelling of the lips and left cheek, left flank pain, and gross hematuria. The patient reported an acute onset of dizziness and headache accompanied by nausea and vomiting 1 week prior to presentation. Her past medical history and family history were unremarkable. On admission, her vital signs were body temperature 36.6℃, blood pressure 140/90 mmHg, heart rate 75 beats per minute, and respiratory rate 20 breaths per minute. Laboratory investigations revealed the following abnormalities: white blood cell count 16.5 × 10^9^/L, hemoglobin 72 g/L, hematocrit 21.1%, granulocyte percentage 78%, Alanine Aminotransferase (ALT) 126 U/L, Aspartate Aminotransferase (AST) 43 U/L, serum potassium concentration 2.54 mmol/L, albumin 28.4 g/L, urinary erythrocyte count 43,608.1 /µl, urinary leukocyte count 415.8 /µl, and D-dimer 12.26 mg/L. Blood coagulation parameters and platelet count were within normal ranges. The erythrocyte sedimentation rate (ESR) was elevated at 34 mm/h (reference range, 0–20 mm/h), and C-reactive protein (CRP) was 23 mg/dL (reference range, 0–6 mg/dL). Doppler ultrasonography demonstrated a 2.5 × 1.2 cm mixed-echoic cystic mass and a systolic color Doppler blood flow signal within the mass near the distal portion of the axillary artery. A parenchymal hematoma was also observed in the left cheek on ultrasound scanning.

**Figure 1:**
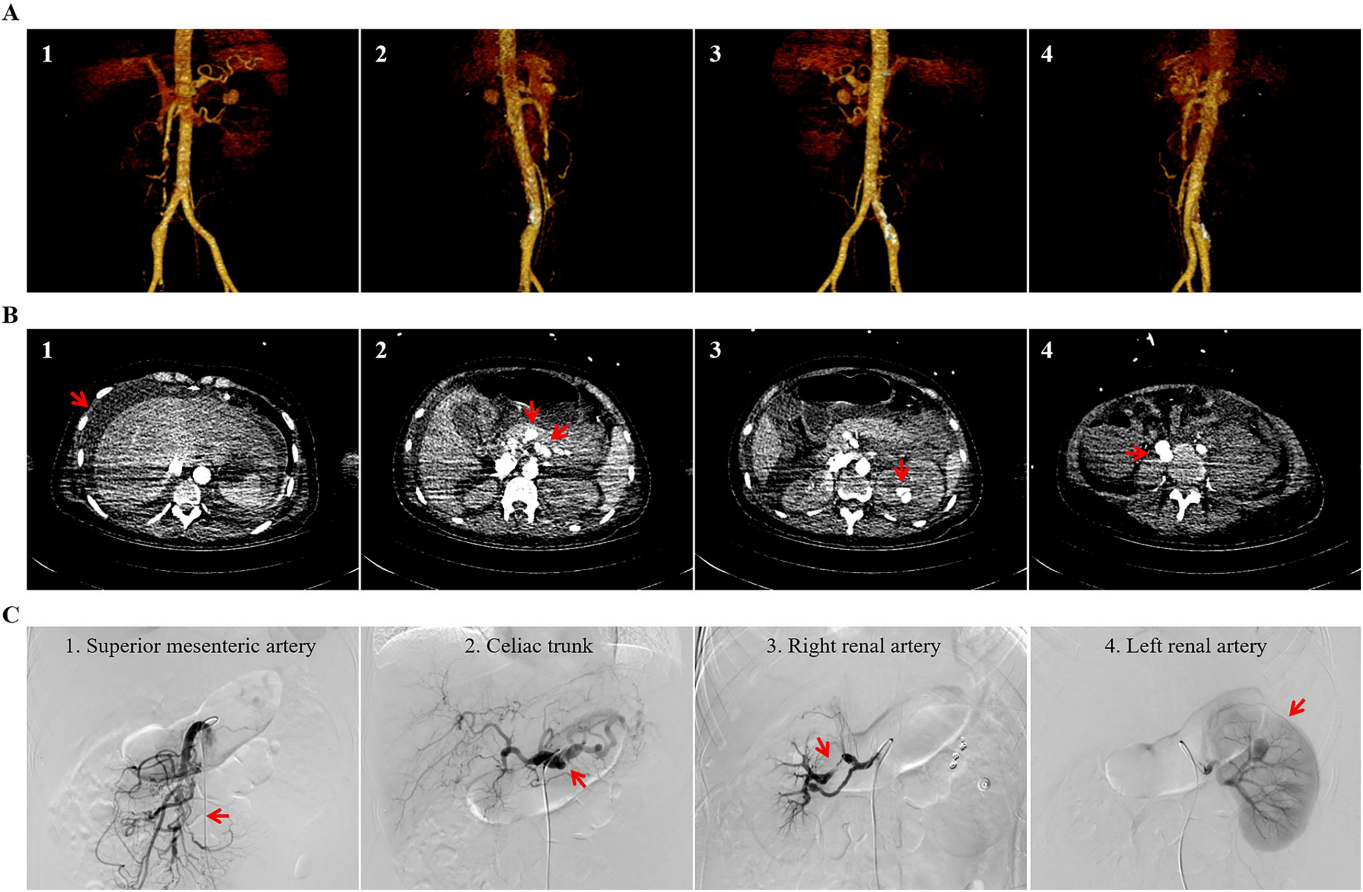
Fibromuscular dysplasia led to renal artery rupture with the involvement of the celiac trunk, splenic, bilateral renal, and superior mesenteric arteries. A CT angiography showed a left renal intraparenchymal hematoma, a common celiac artery aneurysm, and true or false aneurysms alternating with stenosis or occlusion and arterial tortuosity in the celiac trunk, splenic, bilateral renal, and superior mesenteric arteries. The three-dimensional CT angiographic images are in the anterior **(A1)**, right **(A2)**, posterior **(A3)**, and left **(A4)** views. The cross-sectional CT images indicate perihepatic effusion **(B1)**, aneurysm and stenosis in the celiac trunk **(B2)**, renal aneurysm rupture **(B3)**, and aneurysm in the right common iliac artery **(B4)**. Angiographic images of the visceral branch arteries were obtained. The typical “string-of-beads” sign was consistent with fibromuscular dysplasia of the superior mesenteric artery **(C1)**. Angiography shows aneurysms alternating with stenoses in the celiac trunk **(C2)** and right renal artery **(C3)**. Left renal aneurysm rupture was proved by invasive angiography **(C4)** (indicated by arrows).

On the second day after admission, the patient’s white blood cell count and hemoglobin level were 18.68 × 10^9^/L and 68 g/L, respectively. Her respiratory and circulation parameters were stable. On the third day after admission, the patient complained of aggravation of left abdominal pain and sudden-onset fever with a body temperature of 39.6. She also developed signs of shock, including a decline in blood pressure to approximately 90–80/50–40 mmHg, an increase in heart rate to 120–130 beats per minute, and a rise in respiratory rate to 30–40 breaths per minute. Laboratory tests indicated that white blood cell count rose to 41.16 × 10^9^/L, hemoglobin declined to 56 g/L, hematocrit decreased to 15.8%, granulocyte percentage increased to 89.5%, ALT and AST substantially ascended to 5771 and 6,226 U/L, respectively, albumin decreased to 25.3 g/L, creatinine increased from 54.5 to 332.8 µmmol/L, and the D-dimer value was greater than 20 mg/L. The patient received a transfusion of four units of packed red blood cells and 400 mL of plasma. After transfer to the Intensive Care Unit (ICU), the patient underwent endotracheal intubation and mechanical ventilation and received inotropic support (norepinephrine) owing to profound hemodynamic instability. An emergency CT of the brain, chest, abdomen, and pelvis showed a bilateral pleural effusion or hemothorax, predominantly on the right side, extensive and severe left perinephric hemorrhage into retroperitoneal space and pelvic cavity, hematoma in the pancreas–spleen and pancreas–stomach regions, intravesical hematoma, and a suspicious rupture of the bladder wall. A CT angiography of the chest, abdomen, and pelvis revealed a left renal intraparenchymal hematoma measuring 2.1 × 1.5 cm, aneurysmal dilation with a maximum diameter of 1.5 cm in the right common iliac artery, and multiple aneurysms alternating with stenosis, occlusion, and arterial tortuosity in the celiac trunk, splenic, bilateral renal, and superior mesenteric arteries (Figures 1A,B). An invasive angiography confirmed active contrast extravasation from a capsular branch of the left renal artery, as well as multisite dilation, aneurysm, stenosis, occlusion, and tortuosity without contrast extravasation in the celiac trunk, splenic, bilateral renal, and superior mesenteric arteries (Figure 1C). The ruptured artery was successfully embolized using gelfoam and coils. Visceral artery images were compatible with the typical “strings-of-beads” appearance of multifocal FMD. Microbiological cultures of urine were negative for bacteria, but those of blood were positive for *Candida glabrata*. The patient underwent blood transfusion and vein fluid infusion, and she received anti-infective and hepatoprotective drugs as well as continuous renal replacement therapy. During the ICU treatment period, she received a total of six units of red blood cells. A percutaneous abdominal puncture catheter drainage on the right side was performed under ultrasonographic guidance, yielding red blood fluid. Methylene blue was perfused into the bladder thought indwelling catheters. As a result, the blue fluid was drawn out from the abdominal drainage catheter, and the rupture of the bladder wall was further confirmed.

**Figure 2:**
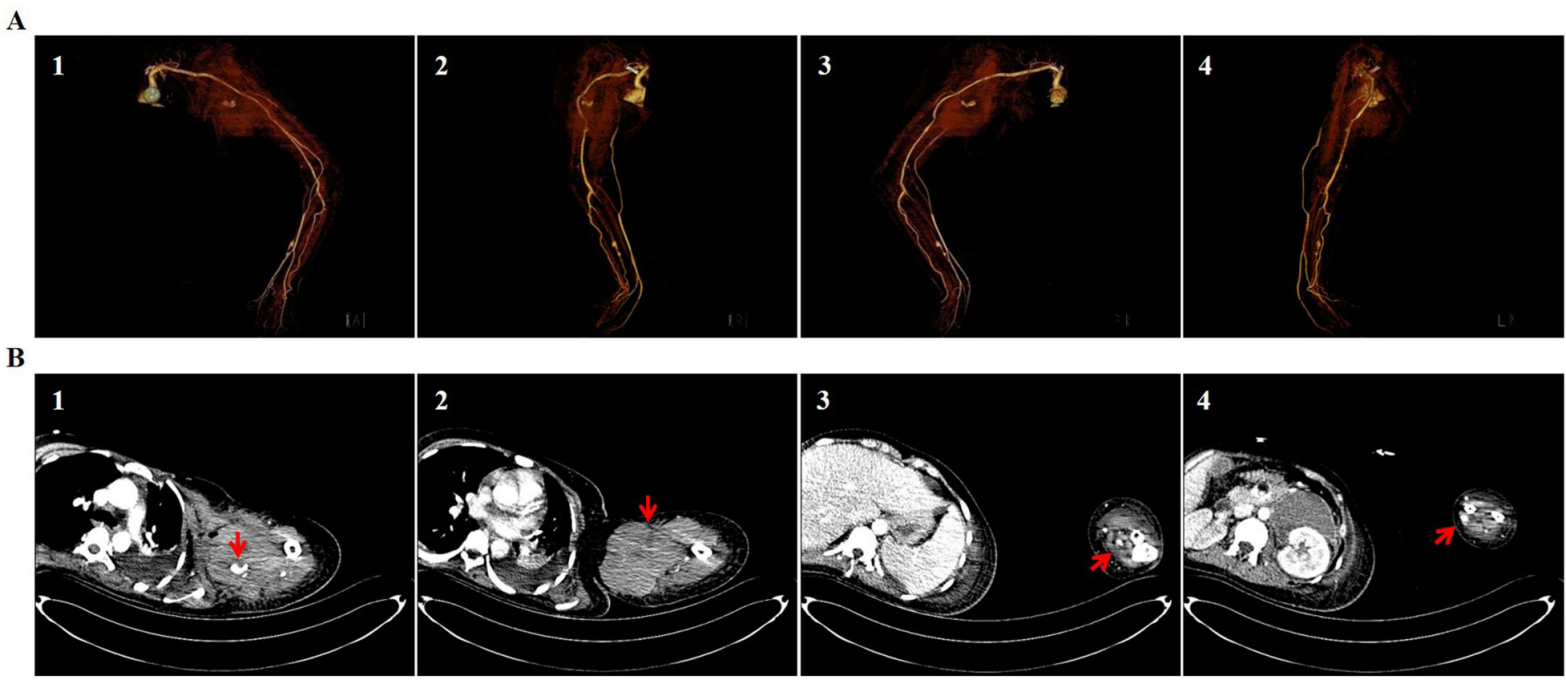
Fibromuscular dysplasia resulted in axillary artery pseudoaneurysm. A CT angiography found soft-tissue hematoma secondary to axillary artery pseudoaneurysm, small proximal brachial artery aneurysm, aneurysms, and stenoses in the proximal radical artery. The three-dimensional CT angiographic images are in the anterior **(A1)**, right **(A2)**, posterior **(A3)**, and left **(A4)** views. The cross-sectional CT images indicate false aneurysm rupture **(B1)**, hematoma **(B2)**, brachial artery aneurysm **(B3)**, and radical artery aneurysm **(B4)** (indicated by arrows).

**Figure 3:**
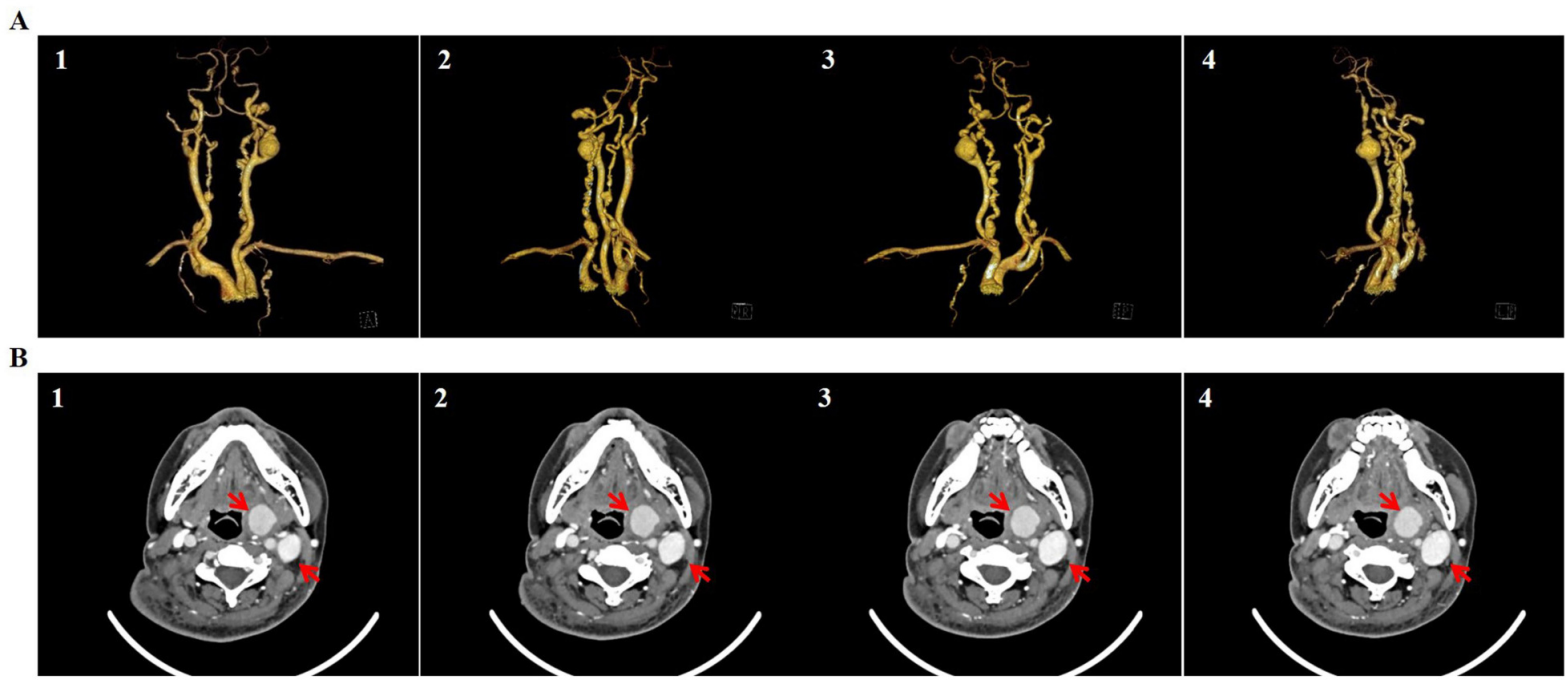
Fibromuscular dysplasia affected the intracranial, vertebral, carotid, and internal mammary arteries. A CT angiography revealed alternating areas of aneurysm and stenosis, described as a “string-of-beads” appearance in the intracranial, vertebral, carotid, and internal mammary arteries. The three-dimensional CT angiographic images are in the anterior **(A1)**, right **(A2)**, posterior **(A3)**, and left **(A4)** views. The cross-sectional CT images indicate a hematoma on the left side of the laryngeal cavity and a large aneurysm in the left internal carotid artery **(B)** (indicated by arrows).

On the 16th day after admission, the patient’s white blood cell count and hemoglobin level were 12.39 × 10^9^/L and 80 g/L, respectively. However, her granulocyte percentage remained elevated at 88.7%. Liver and kidney function parameters recovered to normal ranges. The patient was transferred from the ICU back to the vascular department. However, pain and swelling progressively worsened in the enlarged hematoma in the left upper extremity. An upper-extremity CT angiography showed an extracapsular hematoma measuring approximately 9.6 × 10.2 × 9.8 cm^3^ connected to the distal axillary artery through a thick branch, a 0.5 cm small aneurysm in the proximal brachial artery, two aneurysms with diameters of 0.5 and 0.3 cm in the middle radical artery, and severe narrowing in the proximal radical artery (Figure 2). An intracranial and carotid CT angiography indicated multiple small aneurysms in the anterior and posterior cerebral arteries, a large saccular aneurysm (2.3 × 1.8 cm) in the proximal left internal carotid artery, and a multisite saccular or fusiform aneurysmal dilation alternating with stenosis and tortuosity in the bilateral vertebral, internal carotid, and internal mammary arteries. A 2.1 × 1.8-cm ovoid hematoma with hyperdensity was also noted on the left side of the laryngeal cavity (Figure 3). The multifocal FMD was diagnosed according to the classical strings-of-beads angiographic appearance in the upper-extremity, intracranial, vertebral, carotid, and internal mammary arteries. Open surgery was performed to remove the hematoma and repair the pseudoaneurysm on the 36th day after admission or the 20th day after ICU discharge. The culture of the hematoma sample was positive for *Candida tropicalis*, following which antifungal drugs were administered to the patient. Symptoms of pain and discomfort were significantly alleviated, and her vital and laboratory parameters became relatively stable within four postoperative days.

**Figure 4:**
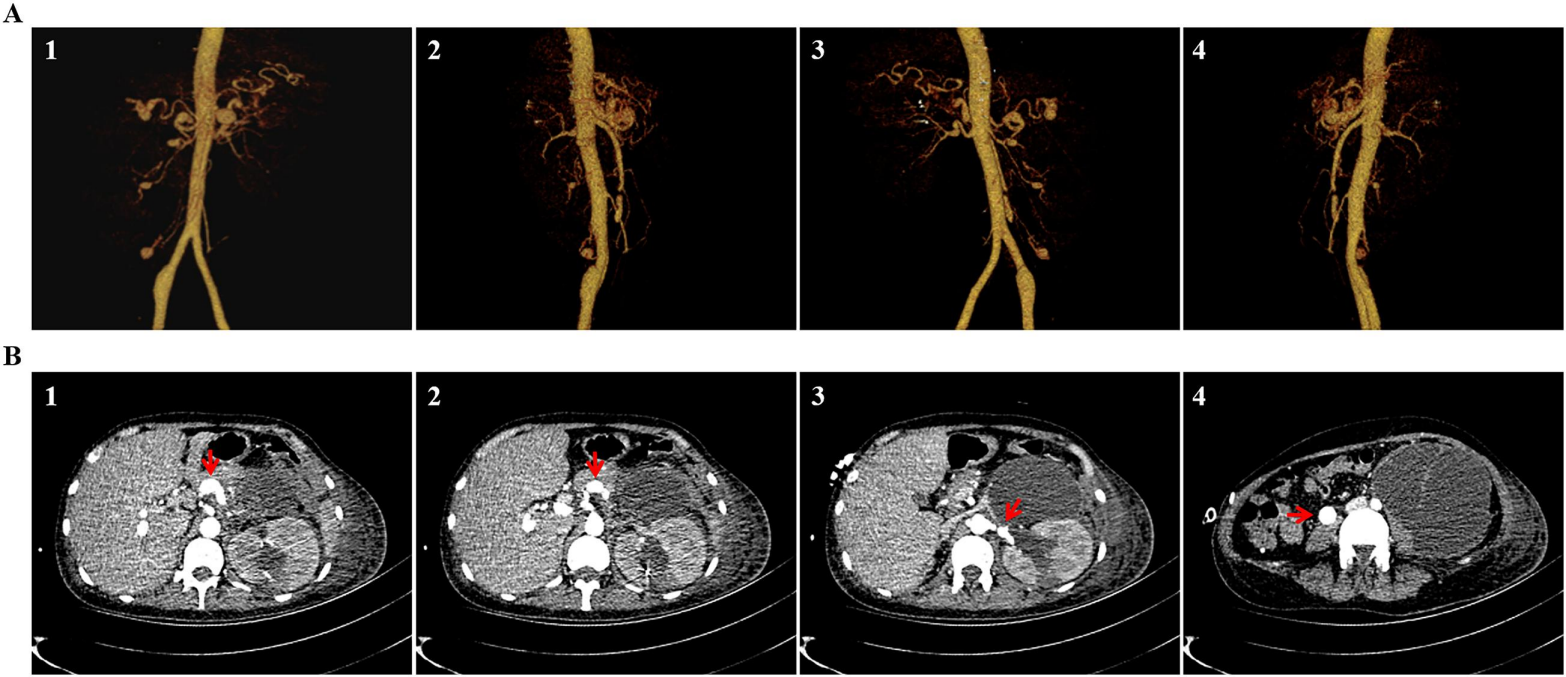
Fibromuscular dysplasia caused recurrent hematoma in the abdominal cavity. A CT angiography indicated an enlarged hematoma on the left of the perinephric space with cystic components and internal septa but no active bleeding site. Fibromuscular dysplasia resulted in stenosis, aneurysm, dissection, occlusion, or arterial tortuosity in the celiac trunk, splenic, bilateral renal, and superior mesenteric arteries. The three-dimensional CT angiographic images are in the anterior **(A1)**, right **(A2)**, posterior **(A3)**, and left **(A4)** views. The cross-sectional CT images indicate a giant hematoma around the left kidney, aneurysm in the celiac artery **(B1)**, celiac artery stenosis **(B2)**, aneurysm and stenosis in the left renal artery **(B3)**, and aneurysm in the right common celiac artery **(B4)** (indicated by arrows).

On the 40th day following admission, the patient complained of severe abdominal distention, predominantly in the left lower abdomen and no exhausting and defecation of the anal. Her vital signs became unstable as follows: a blood pressure of 100–80/50–40 mmHg and heart rate of 100–120 beats per minute. Laboratory testing revealed that hemoglobin declined to 40 g/L and hematocrit dropped to 12%. An emergency CT angiography of the chest, abdomen, and pelvis indicated a left perinephric hematoma around the enlarged left kidney, measuring approximately 10 × 9 × 21 cm^3^, with cystic components, internal septa, and no active arterial bleeding site. Multivessel aneurysms alternating with stenosis and arterial tortuosity in the celiac trunk, splenic, bilateral renal, and superior mesenteric arteries were observed, which were similar to the angiographic findings on admission (Figure 4). These aneurysms manifested as multiple liquid–gas planes indicative of intestinal obstruction and a significant enlargement of peritoneal and pelvic effusion (Figure 4). An abdominal ultrasound demonstrated massive spontaneous hemoperitoneum, divided into multiple compartments with fibrous lamellae, which severely limited the efficacy of the abdominal drainage tube. Two multidisciplinary team discussions were conducted to formulate a treatment strategy and assess the patient's prognosis. The participating specialists included anesthesiologists, interventional radiologists, urologists, gastrointestinal surgeons, and vascular surgeons. A catheter-based invasive angiography was planned for detecting and embolizing potential ruptures of capsular branches or small aneurysms. Exploratory laparotomy with nephrectomy of the left kidney was planned to be done if the blood extravasation site was undetectable or the coil embolism was ineffective. After extensive consultations with her husband and parents, her family expressed concerns about the success of the operation, recurrent aneurysm rupture, life-threatening complications, and long-term ICU stay, eventually declining the invasive endovascular procedure and open surgery. The patient received blood transfusion, intravenous nutrition support, and antifungal treatment. During the last 10 days of hospitalization, she required 12 units of red blood cells. On the 50th day after admission, the symptoms of abdominal distension worsened. The patient became somnolent, and her blood pressure was maintained only with persistent infusion of dopamine. At the request of her husband and parents, the patient was transferred to a local hospital in her hometown, but she died 1 week after discharge.

## Discussion

Historically, FMD has been classified into three histopathological subtypes based on the pathologic layer of the arterial wall, as described by Harrison and McCormack (3). The most common type, medial FMD, presents with alternating areas of thinned media and thickened collagen-contained medial ridges. Its classical “string-of-beads” appearance on angiography is due to alternating areas of stenosis and poststenotic dilatations of the affected artery. Intimal FMD accounts for 5%–10% of cases, with collagen deposition within the intima complicated by a fragmented or duplicated internal elastic lamina. The angiographic appearance of this type is that of a concentric smooth stenosis or long tubular lesion. Adventitial FMD occurs in less than 1% of patients, with dense deposition of collagen in the adventitia resulting in a smooth narrowing appearance on angiography. Because pathology is rarely available in the current era of endovascular intervention, a simplified classification for FMD has been proposed based on the angiographic appearance of the involved arteries (4). Multifocal disease is the most common “string-of-beads” subtype and generally represents the medial FMD. Focal disease resembles circumferential or tubular stenosis and is generally attributed to intimal or adventitial FMD (4).

Symptoms of FMD may vary from a symptomatic condition to multisystem disease, depending on the vascular beds involved and the severity of arterial stenoses. Cerebrovascular FMD may present with transit ischemic attack, stroke, dizziness, neck pain, cervical artery dissection, or pulsatile tinnitus (5). Headache is a common first complaint reported in most patients with cerebrovascular FMD (5). Hypertension is the most common manifestation of renal artery FMD (6, 7). Flank pain may indicate renal artery dissection and infarction, and hematuria occurs in cases of renal aneurysm rupture (6, 7). Hypokalemia due to secondary hyperaldosteronism can also be a presenting feature, but ischemic nephropathy with renal dysfunction is uncommon. FMD involving the extremity arteries may present with intermittent claudication, critical limb ischemia, or peripheral microembolism, manifesting as pain and cyanosis in the toes (8, 9). The most worrisome vascular complications of FMD are true aneurysm rupture, pseudoaneurysm formation, and recurrent catastrophic bleeding events (10). Recent patient cases highlight pseudoaneurysm formation secondary to spontaneous rupture of the axillary arterial branch, a spontaneous rupture of renal artery aneurysm, and a giant intraperitoneal hematoma due to active blood extravasation from the visceral arteries. A profound structural derangement of the true and false aneurysmal wall includes a ruptured internal elastic lamina, multiple tears of the medial layer, and even the absence of smooth muscle layers (11, 12).

Treatment options for FMD vary based on symptoms as well as the nature and location of arterial lesions, but may include medical management, endovascular intervention, and open vascular surgery. There are no definitive treatment guidelines because of histopathological diversity and limited data availability. Antiplatelet or anticoagulation therapy is the standard approach for prevention of thromboembolic events. If there are no contraindications, antiplatelet therapy is more common in patients with a history of coronary artery disease or vascular intervention for FMD. Anticoagulation therapy is often recommended in patients with carotid and vertebral dissection, since the mechanism of stroke is embolization of the thrombus. Antihypertensive medication is important for patients with renal artery involvement and hypertension, and angiotensin-converting enzyme inhibitors or angiotensin receptor blockers are the drugs of choice because FMD is associated with the activation of the rennin–angiotensin–aldosterone system in response to renal ischemia (13). Percutaneous transluminal balloon angioplasty may be considered in patients with renal artery FMD and newly diagnosed hypertension to normalize blood pressure. In addition, angioplasty should be considered in patients with resistant hypertension and those with progressive focal renal artery FMD, which may lead to renal atrophy and decline in renal function. Endovascular stenting may be considered for individuals with refractory stenosis or flow-limiting dissection with ongoing ischemic symptoms (13). The prevalence of aneurysms is high in individuals with FMD. Whether interventional procedures such as surgical clipping and endovascular coiling are necessary must be determined based on the risk of aneurysm rupture. In patients with upper-extremity FMD, surgical bypass has been reported to yield good results, with reversal of ischemia, wound healing, and functional limb salvage (9). Open surgical repair is effective for expanding pseudoaneurysms or for symptomatic cases including hemorrhage and pain.

FMD is an under-recognized, non-atherosclerotic, and non-inflammatory arteriopathy that most commonly affects middle-aged women but may occur across all age groups. FMD may lead to stenosis, aneurysm formation, arterial dissection, occlusion, or tortuosity. FMD can involve multiple, diverse vascular beds and may manifest with a correspondingly wide spectrum of signs and symptoms. Therefore, the second objective of this article is to provide a comprehensive review of clinical diagnosis and management strategies based on the current literature. We present data from 20 published case reports—including their demographic characteristics and clinical outcomes—in Table 1, to enable clinicians to gain a comprehensive understanding of fibromuscular dysplasia (FWD) disease.

**Table 1:** Demographic and clinical characteristics of patients with FMD in published case reports.

Body region	Lesional location	Sex	Age	Clinical manifestation	Treatment modality	CTA characteristics	Authors and references
Head and neck	Right internal carotid artery	M	75	Asymptomatic	Conservative	String of beads	Jahnlova and Veselka (16)
	Carotid and vertebral arteries.	F	26	Neurologic symptoms	No description	Stenosis	Dawley and Ritchie (17)
	Posterior cerebral arteries	F	24	Headache	Conservative	String of beads	Yeo et al. (18)
Upper limb	Right brachial artery	F	68	Asymptomatic	Conservative	String of beads	Miller and Flores (2)
	Right brachial artery	F	76	Cyanosis and pain of the finger	Resection of the brachial artery and saphenous vein graft	Stenosis	Rice and Armstrong (19)
	Right Subclavian Artery	M	20	Asymptomatic	Surgical resection	Aneurysm	Kaneyuki et al. (20)
	Right Subclavian Artery	M	40	Thoracic outlet-like symptoms	Surgical resection	Aneurysm	Stejskal et al. (21)
Abdomen	Right renal artery	F	29	Pain in the right flank and severe hypertension	Conservative	Stenosis and aneurysm	Oliva-Damaso et al. (6)
	Right renal artery	M	28	Resistant hypertension	Balloon angioplasty	Stenosis	Vakili et al. (7)
	Right renal artery	F	49	Abdominal pain	Embolization	Extracapsular hematoma	Akel and Elsayegh (10)
	Splenic artery	M	64	Abdominal pain	Splenectomy	Aneurysm	Watada et al. (11)
	Mesenteric arteries	M	61	Abdominal pain	Conservative	Stenosis and aneurysm	Erwinet al. (12)
	Abdominal aorta	M	77	Moderate intermittent claudication	Bifurcated Dacron graft	Stenosis and aneurysm	Odero et al. (22)
Lower limb	Bilateral iliac artery and right renal artery	M	31	Hypertension	PTA of the renal artery and stent in the iliac artery	Stenosis, aneurysm, and dissection	Fan et al (23)

Abbreviations: CTA, CT angiography; PTA, percutaneous transluminal angioplasty

Tuberculous arteritis is an uncommon condition, usually secondary to the dissemination of mycobacterium tuberculosis infection from the tuberculous tissue to the adjacent arteries (14). Inflammation of the artery may be secondary to direct invasion from the mediastinum, lung, or bone by Mycobacterium tuberculosis (14). True and false aneurysms represent the most common manifestations (15), although stenotic and/or constrictive lesions, along with perivascular fibrosis, have been observed (15). In the case of the patient in this study, ESR and CRP were abnormally elevated, but a CT of the thorax and abdomen did not reveal the presence of pulmonary tuberculosis. Therefore, skin tuberculin testing was not conducted. Given the above, a differential diagnosis of tuberculous arteritis remains a possibility for this patient.

## Limitation

This study reported a severe form of FMD involving multiple vascular beds, including the intracranial, carotid, vertebral, internal mammary, visceral branch, renal, iliac, axillary, and upper extremity arteries. FMD caused adverse outcomes such as axillary artery pseudoaneurysm formation, renal artery aneurysm rupture, left perinephric hematoma, and recurrent catastrophic bleeding events. Two multidisciplinary consensus meetings were convened to determine the optimal treatment modality, involving anesthesiologists, interventional radiologists, urologists, gastrointestinal surgeons, and vascular surgeons. However, the patient's parents and husband ultimately declined the multidisciplinary team's recommendation for endovascular embolization, laparotomy exploration, and left nephrectomy.

## Conclusions

We presented an exceptionally unusual case of a multivessel FMD that resulted in renal artery aneurysm rupture, axillary artery pseudoaneurysm, and catastrophic intraperitoneal hemorrhage. Renal artery rupture was successfully treated with endovascular coil embolization. Open surgical repair was uneventfully performed for axillary artery pseudoaneurysm. Ultimately, the patient experienced catastrophic intraperitoneal hemorrhage and refused endovascular intervention or exploratory laparotomy. This rare and severe FMD affected multiple arterial beds, including the intracranial, carotid, vertebral, internal mammary, visceral branch, renal, iliac, axillary, and upper extremity arteries. While symptomatic FMD is usually suspected in patients, an early CT angiography may expedite diagnosis in patients with otherwise unexplained symptoms of ischemia or hemorrhage.

## Data Availability

The datasets used and/or analyzed during the current study are available from the corresponding author on reasonable request.
